# Effect of Nanopores on Mechanical Properties of the Shape Memory Alloy

**DOI:** 10.3390/mi12050529

**Published:** 2021-05-07

**Authors:** Chunzhi Du, Zhifan Li, Bingfei Liu

**Affiliations:** Aeronautical Engineering College, Civil Aviation University of China, Tianjin 300300, China; czdu@cauc.edu.cn (C.D.); 2019012069@cauc.edu.cn (Z.L.)

**Keywords:** shape memory alloy, nanopores, surface effect, young’s modulus, constitutive model

## Abstract

Nanoporous Shape Memory Alloys (SMA) are widely used in aerospace, military industry, medical and health and other fields. More and more attention has been paid to its mechanical properties. In particular, when the size of the pores is reduced to the nanometer level, the effect of the surface effect of the nanoporous material on the mechanical properties of the SMA will increase sharply, and the residual strain of the SMA material will change with the nanoporosity. In this work, the expression of Young’s modulus of nanopore SMA considering surface effects is first derived, which is a function of nanoporosity and nanopore size. Based on the obtained Young’s modulus, a constitutive model of nanoporous SMA considering residual strain is established. Then, the stress–strain curve of dense SMA based on the new constitutive model is drawn by numerical method. The results are in good agreement with the simulation results in the published literature. Finally, the stress-strain curves of SMA with different nanoporosities are drawn, and it is concluded that the Young’s modulus and strength limit decrease with the increase of nanoporosity.

## 1. Introduction

**Figure 1:**
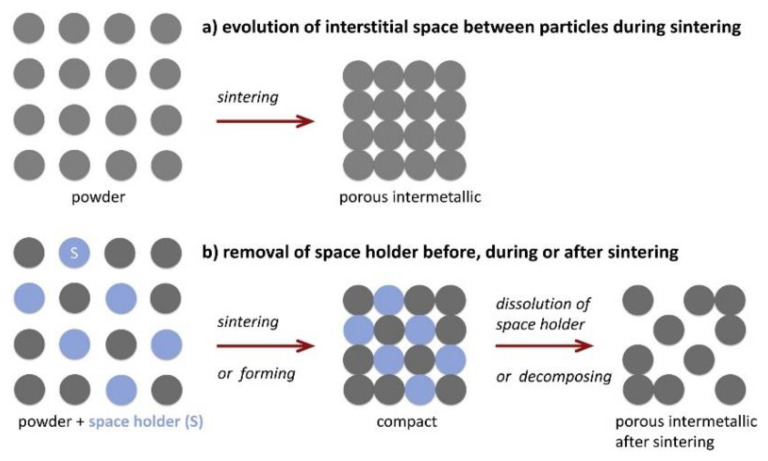
Schematic diagram of the preparation mechanism of porous intermetallic compounds. (**a**) Process of creating porosity (**b**) Process of increasing porosity [5].

Functionally gradient materials and porous materials are new smart materials developed to meet the needs of modern aerospace industries and other high-tech fields [1,2,3,4]. A porous shape memory alloy (SMA) is a porous material with SMA as the frame structure, which can be prepared by powder metallurgy, as shown in Figure 1 [5,6]. It not only has the shape memory effect and super elasticity [7], but also has excellent damping properties and biocompatibility [8]. In general, porous SMA has good performance and can be used in different fields, especially in the medical field.

As the size of the material decreases, the properties of the material will change. For example, microcarbon has higher power storage performance and is often used as a supercapacitor [9,10,11]. Porous materials also have this characteristic. When the porous materials are reduced to the nanoscale, due to the change of the material structure, they will have the characteristics of low density, high specific strength, large specific surface area and low thermal conductivity [12]. The reason is that as the size of the porous material shrinks, the surface effect is enhanced, which has a great impact on the mechanical properties of the porous material [13].

Research into the macro-mechanical properties of nanoporous materials with interfacial effects has attracted more and more attention from scholars at home and abroad in recent years. Sharma predicted that the effective elastic modulus of nanoporous materials depends on the size of pores [14,15]. Xun et al. had studied the effective elastic modulus of micro polarized materials with inhomogeneous spherical and cylindrical pores and found that the effective elastic modulus of micro-polarized materials depends on the size of inhomogeneous pores [16]. Duan et al. had established a general micromechanical model considering interface effect [17,18]. Quang et al. developed the minimum potential energy and complementary energy theorems of linear elasticity for inhomogeneous materials with interface effect [19]. Feng et al. estimated the effective modulus of perforated material [20]. Xia studied the macroscopic mechanical properties of open-ended nanoporous materials with surface elasticity and surface residual stress [21].

Most of the above studies are about porous carbon or single metal nanoporous materials, but the research on nanoporous SMA is rarely mentioned, especially the effect of size on material properties. In view of this, the relationship between the mechanical properties of SMA materials with nanopores and the surface effects, especially the relationship between the mechanical properties of SMA materials and different nanopore diameters or nanoporosities, respectively, is discussed in this work. Combining the framework of micromechanics theory including surface effect, firstly, the effective Young’s modulus of nanoporous SMA is calculated by composite material equivalent method. A constitutive model of SMA with nanopores considering surface effect and residual strain is then established.

## 2. Young’s Modulus of SMA with Nanopores

When the size of the material reaches the nanometer level, the surface effect needs to be considered. The stress corresponding to the surface effect is the surface stress, which can be defined in many ways. Nix et al. proposed an atomic interpretation of surface/interface stress, in which the relationship between surface stress and strain is attributed to the change in the bonding environment of atomic bonds [22]. Bottomley and Ogino proposed a linear expression between surface stress tensor τ and surface elastic strain tensor $\varepsilon^{s}$ [23], which is called Hooke’s law in their paper

(1) $$\tau =C^{s}:\varepsilon^{s}.\;\;\;\;\;\;\;$$

where $C^{s}$ is the surface stiffness tensor. When considering material isotropy, constitutive Equation (1) can be simplified to the following

(2) $$\tau =\lambda_{s}\left(tr\varepsilon^{s}\right)I^{\left(2\right)}+2\mu_{s}\varepsilon^{s}.\;\;$$

where $\lambda_{s}$ and $\mu_{s}$ are the surface modulus, and $I^{\left(2\right)}$ is the second-order unit tensor in two-dimensional space.

Sun et al. pointed out that when considering the change of the stress field, the effective elastic properties will be affected by the surface stress [24]. Therefore, surface stress must be taken into consideration when calculating the elasticity of nanomaterials. Duan et al. derived the effective bulk modulus and effective shear modulus of the composite containing nano-inhomogeneous inclusion on the basis of the micromechanical framework [17,18]. The composite material with nano-inhomogeneous inclusion consists of an inclusion phase and a matrix phase, and there is surface stress between the surfaces of the two phases. The bulk modulus of the composite material $\kappa_{3}$ is composed of the bulk modulus $\kappa_{1}$ of the nano-inhomogeneous inclusion phase and the bulk modulus $\kappa_{2}$ of the matrix. The shear modulus of the composite material $\mu_{3}$ is composed of the shear modulus $\mu_{1}$ of the nano-inhomogeneous inclusion phase and the shear modulus $\mu_{2}$ of the matrix. f and 1−f denote the volume fraction of the nano-inhomogeneous inclusion phase and matrix, respectively. The expressions of the bulk modulus $\kappa_{3}$ and shear modulus $\mu_{3}$ of the composite material with nano-inhomogeneous inclusion deduced by Duan are as follows [18]

(3) $$\kappa_{3}=\frac{3\kappa_{1}\left(3\kappa_{2}+4f\mu_{2}\right)+2\mu_{2}\left[4f\mu_{2}\kappa_{s}^{r}+3\kappa_{2}\left(2-2f+\kappa_{s}^{r}\right)\right]}{3\left[3\left(1-f\right)\kappa_{1}+3f\kappa_{2}+2\mu_{2}\left(2+\kappa_{s}^{r}-f\kappa_{s}^{r}\right)\right]}.$$

where $\kappa_{s}^{r}=\kappa_{s}/\left(R_{0}\mu_{2}\right)$, $\kappa_{s}=2\left(\mu_{s}+\lambda_{s}\right),\;R_{0}$ is the size of the nano-inhomogeneous inclusion phase.

(4) $$\mu_{3}=\frac{\mu_{2}\left[5-8f\xi_{3}\left(7-5v_{2}\right)\right]}{5-f\left(5-84\xi_{1}-20\xi_{2}\right)}.$$

where

(5) $$\xi_{1}=\frac{15\left(1-v_{2}\right)\left(\kappa_{s}^{r}+2\mu_{s}^{r}\right)}{4H}.$$

(6) $$\xi_{2}=\frac{-15\left(1-v_{2}\right)}{4H}\left[\eta_{1}\left(7+5v_{1}\right)-8v_{1}\left(5+3\kappa_{s}^{r}+\mu_{s}^{r}\right)+7\left(4+3\kappa_{s}^{r}+2\mu_{s}^{r}\right)\right].$$

(7) $$\begin{array}{c} \xi_{3}=\frac{5}{16H}\left[2\eta_{1}^{2}\left(7+5v_{1}\right)-4\left(7-10v_{1}\right)\left(2+\kappa_{s}^{r}\right)\left(1-\mu_{s}^{r}\right)\right]\\ \;\;\;\;\;+\frac{5}{16H}\eta_{1}\left[7\left(6+5\kappa_{s}^{r}+4\mu_{s}^{r}\right)-v_{1}\left(90+47\kappa_{s}^{r}+4\mu_{s}^{r}\right)\right]. \end{array}$$

(8) $$\begin{array}{ll} H=-2\eta_{1}^{2}(7+ & 5v_{1})\left(4-5v_{2}\right)+7\eta_{1}\left[-39-20\kappa_{s}^{r}-16\mu_{s}^{r}+5v_{2}\left(9+5\kappa_{s}^{r}+4\mu_{s}^{r}\right)\right]\\ & +\eta_{1}v_{1}\left[285+188\kappa_{s}^{r}+16\mu_{s}^{r}-5v_{2}\left(75+47\kappa_{s}^{r}+4\mu_{s}^{r}\right)\right]\;\\ & +4\left(7-10v_{1}\right)\left\{-7-11\mu_{s}^{r}-\kappa_{s}^{r}\left(5+4\mu_{s}^{r}\right)\right\}.\; \end{array}$$

with $\mu_{s}^{r}=\mu_{s}/\left(R_{0}\mu_{2}\right)$, and $\;\eta_{1}=\mu_{1}/\mu_{2}$. $v_{1}$ and $v_{2}$ represent the Poisson’s ratio of the inclusion phase and the matrix phase, respectively. The subscript s indicates physical quantities related to the surface. The effective modulus of composite materials with nano-inhomogeneous inclusion depends on two dimensionless parameters $\kappa_{s}^{r}$ and $\mu_{s}^{r}$.

Applying the above theory to nanoporous SMA materials, using SMA as the matrix, and degenerating the nano-inhomogeneous inclusion into nanopores, i.e., ($\mu_{1}=\kappa_{1}=0,\;v_{1}=0$), the elasticity modulus of nanoporous SMA is derived using the micromechanical framework

(9) $$\kappa_{np}=\frac{2\mu_{SMA}\left[4f\mu_{SMA}\kappa_{s}^{r}+3\kappa_{SMA}\left(2-2f+\kappa_{s}^{r}\right)\right]}{3\left[3f\kappa_{SMA}+2\mu_{SMA}\left(2+\kappa_{s}^{r}-f\kappa_{s}^{r}\right)\right]}.$$

(10) $$\mu_{np}=\frac{\mu_{SMA}\left[5H+70f\left(7-5v_{SMA}\right)\left(2+\kappa_{s}^{r}\right)\left(1-\mu_{s}^{r}\right)\right]}{5H-f\left(1-v_{SMA}\right)\left(5H+1260\kappa_{s}^{r}+420\mu_{s}^{r}+2100\right)}.$$

where

(11) $$\kappa_{s}^{r}=\kappa_{s}/\left(R\mu_{SMA}\right).$$

(12) $$\kappa_{s}=2\left(\mu_{s}+\lambda_{s}\right).$$

(13) $$H=28\left\{-7-11\mu_{s}^{r}-\kappa_{s}^{r}\left(5+4\mu_{s}^{r}\right)+v_{\mathrm{SMA}}\left[5+13\mu_{s}^{r}+\kappa_{s}^{r}\left(4+5\mu_{s}^{r}\right)\right]\right\}.\;$$

(14) $$\mu_{s}^{r}=\mu_{s}/\left(R_{0}\mu_{SMA}\right).$$

There are four unknown constants and two unknown quantities R,  f. The constants $\lambda_{s}$, $\;\mu_{s}$,$\;\;\kappa_{2}$, and $\;\mu_{2}$ need to be determined. The bulk modulus and shear modulus of SMA are as follows,

(15) $$\mu_{SMA}=\frac{E_{SMA}}{2\left(1+\nu_{SMA}\right)}.$$

(16) $$\kappa_{SMA}=\frac{E_{SMA}}{3\left(1-2\nu_{SMA}\right)}.$$

where

(17) $$E_{SMA}=E^{A}+\xi \left(E^{M}-E^{A}\right).\;$$

where $E^{A}$, $\;E^{M}$ are the Young’s modulus of 100% austenite and 100% martensite phases for dense SMA, respectively. The ξ is the martensitic volume fraction. Suppose that the Poisson’s ratio $\nu_{SMA}$ of SMA does not change during the deformation process, and ξ is a linear function of applied stress, which is slightly different from popular models [25].

(18) $$\xi =\{\begin{array}{c} 0\;\;\;\;\;\;\;\;\;\;\;\;\;\;\;\;\;\;\;\;\;\;\;\sigma \leq \sigma_{s}\;\;\;\\ \frac{\sigma -\sigma_{s}}{\sigma_{f}-\sigma_{s}}\;\;\;\;\;\;\;\;\;\;\;\;\sigma_{s}\;\leq \sigma \leq \sigma_{f}.\;\\ 1\;\;\;\;\;\;\;\;\;\;\;\;\;\;\;\;\;\;\;\;\;\sigma_{f}\leq \;\sigma \;\;\;\; \end{array}\;\;\;\;\;\;\;\;\;\;\;\;\;\;\;\;\;\;\;\;\;\;$$

where σ is the effective stress and $\sigma_{s},\;\sigma_{f}$ are the threshold stress at the beginning and end of the SMA transition, respectively. The martensitic volume fraction ξ can be easily obtained once the threshold stresses of the porous SMA are determined. 

However, it is difficult to measure the elasticity Lame constants $\lambda_{s}$ and $\mu_{s}$ of the surface of SMA. By linking the surface Lame constants $\lambda_{s}$ and $\mu_{s}$ with the overall Lame constants $\lambda_{SMA}$ and $\;\mu_{SMA}$, the overall Lame constants can be assumed to be an integer multiple of the surface Lame constants, namely

(19) $$\lambda_{s}=\lambda_{SMA}/c_{1}.\;$$

(20) $$\mu_{s}=\mu_{\mathrm{SMA}}/c_{2}.$$

where

(21) $$\lambda_{SMA}=\frac{\nu_{SMA}E_{SMA}}{\left(1+\nu_{SMA}\right)\left(1-2\nu_{SMA}\right)}.$$

Then the Young’s modulus $E_{np}$ of SMA with nanopores is obtained from the bulk modulus $\kappa_{np}$ and the shear modulus $\mu_{np}$,

(22) $$E_{np}=\frac{9\kappa_{np}\mu_{np}}{3\kappa_{np}+\mu_{np}}.$$

## 3. Constitutive Model of SMA with Nanopores Considering Surface Effect and Residual Strain

### 3.1. Residual Strain of SMA with Nanopores

Residual strain refers to the strain that the material cannot return to its original state after being unloaded. Shape memory alloys with large porosity exhibit greater macroscopic residual strain. It has been found that the residual strain increases approximately linearly with the increase of porosity by Sourav Gur et al. [26]. In order to obtain an analytical solution, the residual strain can be assumed to be a linear equation of porosity. For the residual strain $\varepsilon_{np}^{r}$ of nanoporous SMA, it is assumed that $\varepsilon_{np}^{r}$ is a linear function of nano porosity.

(23) $$\varepsilon_{np}^{r}=\left(1+f\right)\varepsilon_{ns}^{r}.\;$$

where the residual strain $\varepsilon_{ns}^{r}$ of the nano-scale dense SMA can be obtained by simulation. 

### 3.2. Constitutive Model of SMA with Nanopores

The research on the constitutive model of SMA has been fully developed in recent decades, and it has been reported in detail in theoretical research and experiments [27,28]. In this paper, the following one-dimensional constitutive model of SMA is selected as an example for analysis [29].

(24) $$\varepsilon =\frac{\sigma }{E^{A}+\xi \left(E^{M}-E^{A}\right)}+\alpha \left(T-T_{0}\right)+H\xi +\varepsilon^{r}.\;$$

where ε, σ, α are total strain, stress and thermal expansion coefficient, T is temperature and $T_{0}$ is the temperature in the initial state, H is the largest phase transformation strain, $\varepsilon^{r}$ is the residual strain, can be obtained from simulation or experimental data.

For SMA with nanopores, the constitutive model considering the surface effect and the porosity of nanopores can be expressed as follows:

(25) $$\varepsilon_{np}=\frac{\sigma }{E_{np}}+\alpha_{np}\left(T-T_{0}\right)+H\xi_{np}+\varepsilon_{np}^{r}.\;$$

where

(26) $$\xi_{np}=\{\begin{array}{c} 0\;\;\;\;\;\;\;\;\;\;\;\;\;\;\;\;\;\;\;\;\;\;\;\sigma \leq \sigma_{s}^{n}\;\;\;\\ \frac{\sigma -\sigma_{s}^{np}}{\sigma_{f}^{np}-\sigma_{s}^{np}}\;\;\;\;\;\;\;\;\;\;\;\;\sigma_{s}^{n}\;\;\leq \sigma \leq \sigma_{f}^{n}\;\;\\ 1\;\;\;\;\;\;\;\;\;\;\;\;\;\;\;\;\;\;\;\;\;\sigma_{f}^{n}\leq \;\sigma \;\;\;\; \end{array}.$$

with σ is stress, $\sigma_{s}^{np}$, $\sigma_{f}^{np}$ are the threshold stress at the beginning and end of the nanoporous SMA transformation, and $\sigma_{s}^{ns}$ and $\sigma_{f}^{ns}$ are threshold stress at the beginning and end of the transformation of the dense SMA, respectively. The superscript and subscript np are used to indicate the nanoporous SMA. Shape memory alloys with larger porosity exhibit smaller threshold stresses at the beginning and end of the transformation. According to reference [26], it has been found that the threshold stresses decrease approximately linearly with the increase of porosity. In order to obtain an analytical solution, the threshold stresses can be assumed to be a linear equation of porosity.

(27) $$\{\begin{array}{c} \sigma_{s}^{np}=\left(1-f\right)\sigma_{s}^{ns}\\ \sigma_{f}^{np}=\left(1-f\right)\sigma_{f}^{ns} \end{array}.$$

## 4. Numerical Results

### 4.1. Numerical Results of the Young’s Modulus SMA with Nanopores

In order to obtain the analytical solution of the model in this paper, the surface elastic constants of aluminum are assumed to be the surface elastic constants of nanoporous SMA. The surface elastic constants of aluminum are taken from the papers of Miller and Shenoy, in which two sets of surface moduli are used, namely,$\;\kappa_{s}=-5.457\;N/m,$$\mu_{s}=-6.2718\;N/m$ for surface <100>; $\kappa_{s}=12.932\;N/m,\;\mu_{s}=-0.3755\;N/m$ for surface <111>. The ratio coefficient can be obtained as follow $c_{1}^{1}=14.92,c_{2}^{1}=-5.58$ and $c_{1}^{2}=8.9527,c_{2}^{2}=-92.41$ [30]. The numerical results of SMA were given by Lagoudas D C, et al. ($E^{M}=30\mathrm{GPa},\;E^{A}=70\mathrm{GPa},\;v_{SMA}=0.3$ [31]).

**Figure 2:**
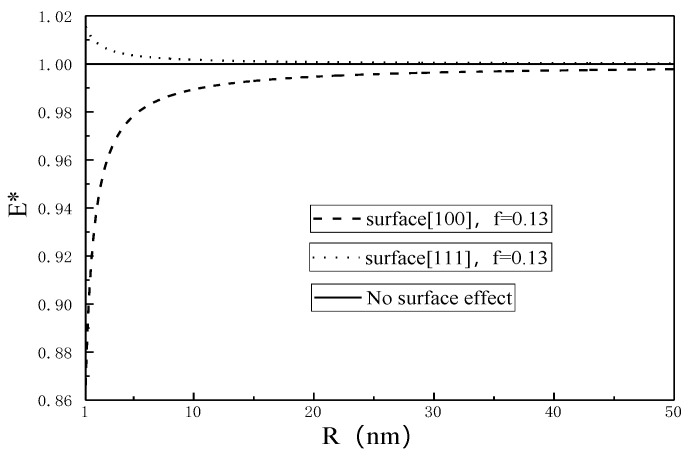
Effective Young’s modulus of 100% martensite as a function of nanopore radius.

**Figure 3:**
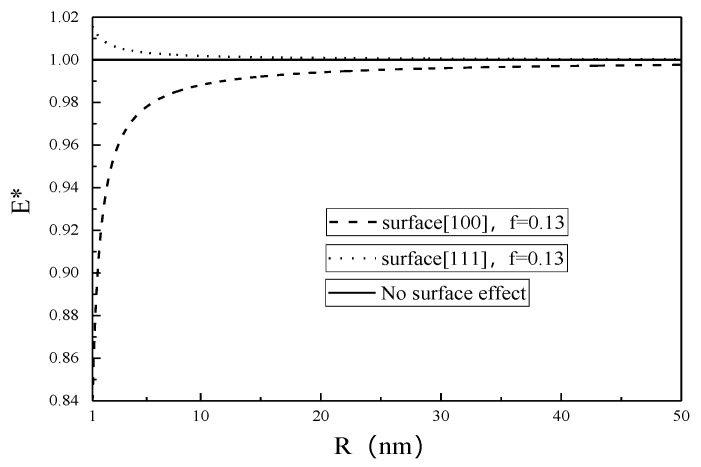
Effective Young’s modulus of 100% austenite as a function of nanopore radius.

For brevity, define $E^{*}=E_{np}/E$, where E represents the classical results without the surface effect ($\kappa_{s}=\mu_{s}=0$). The normalized Young’s modulus $E^{*}$ for different surface properties as a function of nanopore radius is plotted in Figure 2 and Figure 3.

It is seen from Figure 2 and Figure 3 that $E^{*}$ decreases (increases) with the increase of nanopore size due to the surface effect, while the classical result (without the surface effect) is independent of the nanopore size. When R is close to 50 nm, the Young’s modulus of SMA with nanopores is in good agreement with that of porous materials obtained by Zhao [32]. The surface effect on the Young’s modulus becomes negligible for nanopore radius larger than 50 nm.

**Figure 4:**
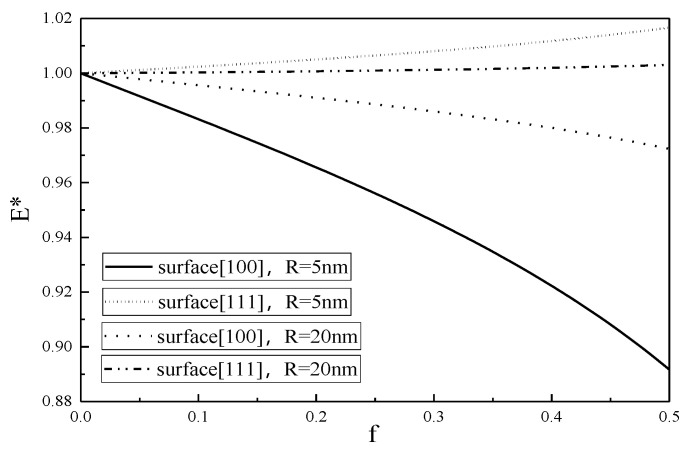
Effective Young’s modulus of 100% martensite as a function of nanoporosity.

**Figure 5:**
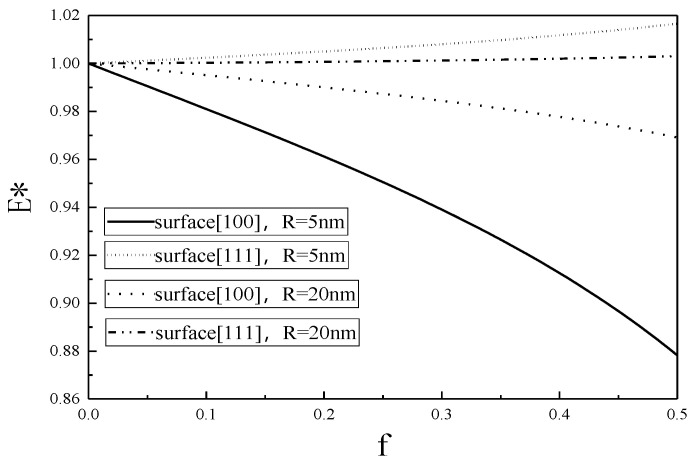
Effective Young’s modulus of 100% austenite as a function of nanoporosity.

The variations of the Young’s modulus $E^{*}$ with nano porosity f for two different nanopore radius are shown in Figure 4 and Figure 5.

The value of $E^{*}$ represents a deviation from the classical result without the surface effect, and it is seen from Figure 4 and Figure 5 that the smaller the nanopore, the larger the deviation, showing that the smaller the nanopore, the more obvious the surface effect.

**Figure 6:**
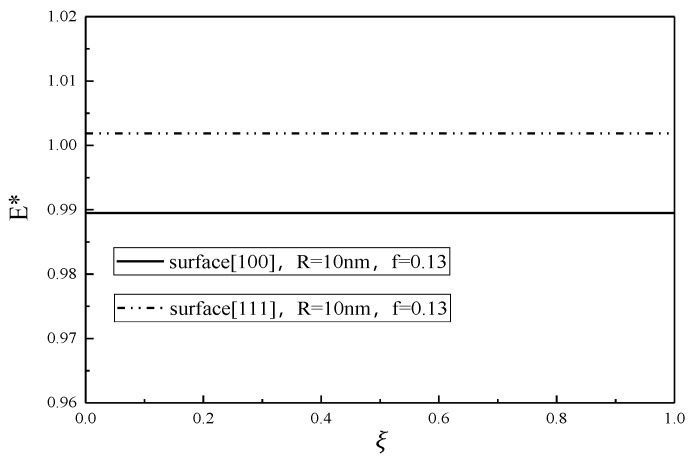
Effective Young’s modulus of nanoporous SMA as a function of the martensite volume fraction ξ.

The relationship between the normalized Young’s modulus $E^{*}$ and the volume fraction ξ of martensite at different transformation stages is shown in Figure 6. It is seen that the effective Young’s modulus of nanoporous SMA is independent of the volume fraction of martensite.

From Figure 1, Figure 2, Figure 3, Figure 4, Figure 5 and Figure 6, it is seen that the effective Young’s modulus increases for the surface <100>, while for the surface <111>, the effective Young’s modulus decreases. The surface effect has a significant effect on the effective Young’s modulus of the SMA with nanopores on the surface <100>, but has no obvious effect on the effective Young’s modulus of the SMA with nanopores on the surface <111>.

### 4.2. Numerical Results of the Constitutive Model

**Figure 7:**
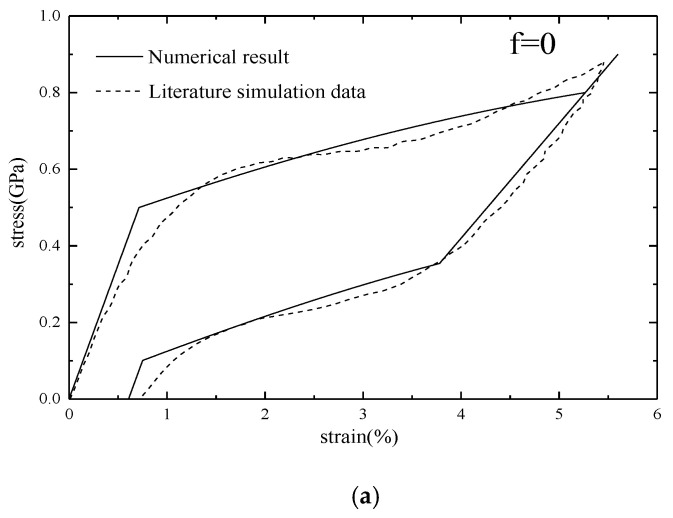

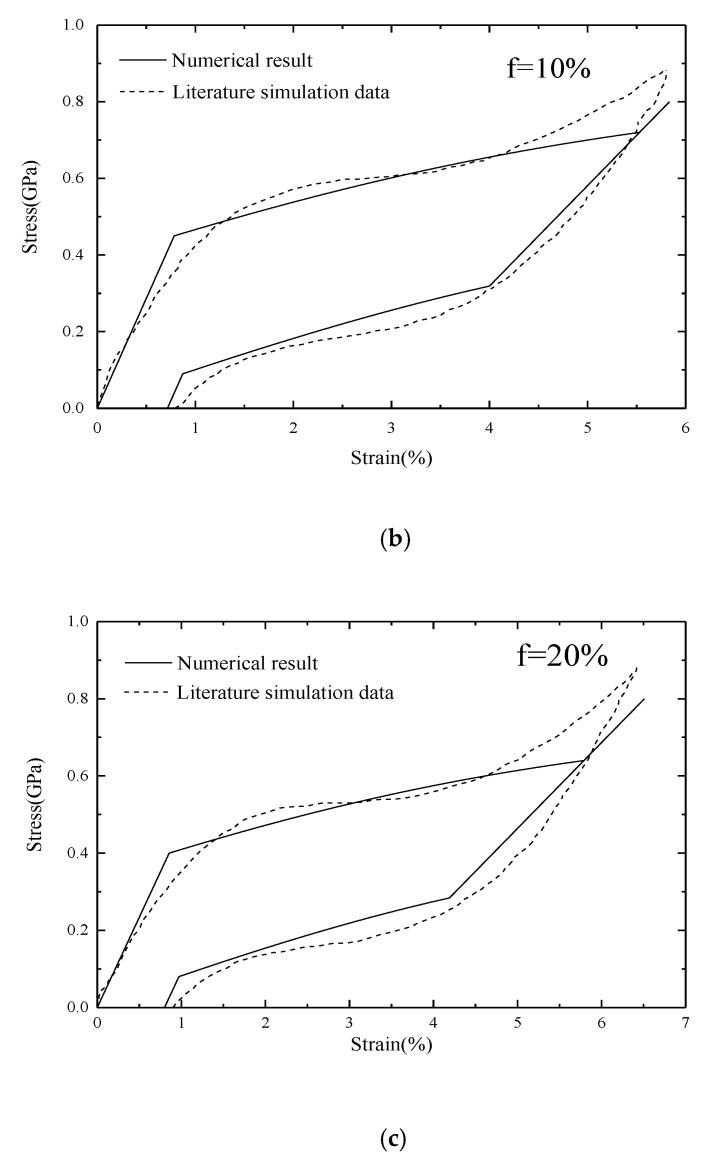
The stress-strain curves of simulation data and numerical result of (**a**) non-porous NiTi, (**b**) 10% porosity NiTi and (**c**) 20% porosity NiTi [32].

In this paper, the effective Young’s modulus of SMA with nanopores can be obtained. Gur et al. established a nanoporous NiTi shape memory alloy model with different porosities. The initial size of the model is 250 Å × 250 Å × 250 Å, and the pore size is 10–50 Å. And through molecular dynamics simulation, the stress-strain curves of NiTi shape memory alloys with different porosities were obtained [26], from which the threshold stresses of SMA with different porosities can be extracted. In the MD simulation of the cited reference [26], the <100> and <110> crystal orientation models of the NiTi porous shape memory alloy were simulated. While, the Young’s moduli of the NiTi porous shape memory alloy in the <100> and <111> crystal orientations are only obtained in this paper. In order to verify the correctness of the model in this paper, the <100> crystal orientation is used for numerical calculation and compared with the Gur’s simulation data. It can be seen from Figure 7 that there is a high degree of agreement between the simulation data and the numerical result.

## 5. Conclusions

In this work, based on the micromechanical framework and considering the surface effect, the effective Young’s modulus of the SMA with nanopores is obtained. Based on Young’s modulus and residual strain, a constitutive model of nanoporous SMA is established. In the numerical results, the effective Young’s modulus is plotted against nanopore radius, nanoporosity and martensite volume fraction, and the stress–strain curves of SMAs with different nanoporosities are plotted.

1. The effective Young’s modulus of nanoporous SMA, which is independent of the volume fraction of martensite, depends on the radius of nanopores and nanoporosity.
2. The surface effect decreases with the increase of nano pore size, and the effective Young’s modulus of nanoporous SMA is close to the macroscopic theory when the nano pore radius exceeds 50 nm.
3. The constitutive model of nanoporous SMA is derived, which is in good agreement with the simulation data in the literature.

## Data Availability

All data generated or analyzed during this study are included in the manuscript. All the raw data sets generated or analyzed during the current study are available from the corresponding author on reasonable request.
